# Empirical analysis of spatial heterogeneity in the development of China’s National Fitness Plan

**DOI:** 10.1371/journal.pone.0305397

**Published:** 2024-06-13

**Authors:** Yuanbo Hu

**Affiliations:** International College, Krirk University, Bangkok, Thailand; Arba Minch University, ETHIOPIA

## Abstract

**Purpose:**

The National Fitness Plan (NFP) is a vital initiative aimed at realizing Healthy China 2030. This study assessed spatial heterogeneity in the NFP development and the socioeconomic factors contributing to this inequality.

**Methods:**

Data from 31 administrative regions in 2021 were analyzed using four NFP development metrics. Spatial autocorrelation was evaluated using global Moran’s *I*, followed by global and local regression models for non-random spatial patterns.

**Results:**

National physical fitness exhibited significant clustering (z = 5.403), notably a high-high cluster in East China. The global regression model identified three socioeconomic factors in the geographically weighted regression model: per capita disposable income and the number of public buses positively affected national physical fitness, while general public budget expenditure had a negative impact.

**Conclusions:**

Persistent unequal NFP development is projected due to income disparities in economically backward regions. To promote the NFP effectively, a cost-efficient strategy includes creating 15-minute fitness circles, especially by establishing public sports facilities in Western China communities. These findings inform policy priorities for advancing the NFP towards Healthy China 2030.

## 1. Introduction

In 2021, the National Development and Reform Commission issued the “the Outline of the 14th Five-Year Plan (2021–2025) for National Economic and Social Development and Vision 2035 of the People’s Republic of China” (thereafter, 14th Five-Year Plan) [1]. This plan underscores the prioritization of improving public health and advancing the sports industry to achieve Healthy China 2030. Needless to say, the promotion of high-quality fitness-for-all campaigns is a crucial objective of the 14th Five-Year Plan, integral to the national modernization [2]. Since the implementation of the National Fitness Program Outline in 1995, China has made initial progress in enhancing public health through sports. According to the National Physical Fitness Monitoring Bulletin, 37.2% of individuals aged seven and above engaged in regular physical activity in 2020 [3]. Meanwhile, there remain several shortcomings in establishing all-around public services for national fitness. The latest National Fitness Plan (NFP) acknowledges “problems such as the unbalanced development of the national fitness area and the insufficient supply of public services still exist” [4], highlighting key objectives for the NFP going forward. Therefore, investigating regional imbalances and underlying mechanisms holds immense theoretical and practical importance in attaining a superior level of Healthy China 2030.

Many studies have provided valuable recommendations to facilitate the high-quality development of the NFP. For example, Han’s analysis of the NFP (2016–2020) across 31 administrative regions revealed that current NFP-related public policies often lack sport-specific guidelines due to reliance on conventional public administration methods [5]. Based on a review of the NFP’s achievements and shortcomings during the 13th Five-Year Plan, Lu and Wang have proposed a set of policy suggestions for the NFP [6], including developing an advanced public sports service system. The primary gap in policy recommendations for the NFP development, compared to existing research, is the predominance of qualitative reviews focusing on macro environments and policy decisions. This leads to a lack of quantitative research that can objectively identify the NFP’s strengths and weaknesses, especially regarding regional imbalances [4]. For instance, the latest NFP sets a goal of having 2.16 social sports instructors per 1,000 people by 2025, but it’s unclear if there’s a regional imbalance in meeting this target. Addressing this requires more quantitative studies, particularly employing geographical analysis methods.

The multifaceted nature of the NFP poses challenges in evaluating its effectiveness and understanding the factors influencing its development. Geographical research has predominantly focused on two aspects of the NFP: spatial disparities in public sports venues and facilities, and spatial heterogeneity in national physical fitness. First, there appear to be spatial disparities in public sports venues and facilities. Wei et al. conducted a spatial-temporal analysis spanning 1995 to 2013, revealing significant spatial autocorrelation in the numbers of public sports venues and facilities, with regions of high density aligning with stronger provincial economies [7]. Similarly, Song et al., using Moran’s *I* and cluster analysis in 2014, found fewer public sports venues and facilities in rural areas compared to urban regions, indicating economic influences on this geographical disparity [8]. Second, research on national physical fitness across various years (2005, 2010, 2014, and 2015) has consistently demonstrated significant heterogeneity among the 31 administrative regions [9–12].

There are two critical shortcomings in the existing geographic research on the NFP. First, most studies provide data only up to 2015, making policy evaluation for the NFP’s regional development in the 2020s uncertain and potentially less useful. Second, whereas traditional geographical analyses such as Moran’s *I* can detect spatial heterogeneity, they do not explain the underlying mechanisms. In this regard, only one group of researchers has delved into the factors contributing to geographic heterogeneity. In their three separate studies on the provincial-level reports of national physical fitness in 2015, Wang and Hu identified environmental variables and per capita regional GDP as primary determinants [10–12]. However, these studies have not examined various socioeconomic factors, such as the correlation between accessibility of public sports venues and facilities and individuals’ physical activity time [13], which directly influences physical fitness. Hence, it is necessary to conduct a broad analysis of various socioeconomic factors related to the NFP.

Therefore, the purpose of this study was to explore spatial heterogeneity and socioeconomic factors contributing to the NFP’s unequal development. By identifying key socioeconomic determinants, this study aims to establish a theoretical foundation for the coordinated development of the NFP.

## 2. Methods

### 2.1 Evaluation variables

Based on the premise of using the most recent data available, this study analyzed the 2021 data from the 31 administrative regions of China Mainland. According to the eight developmental goals outlined in the NFP [4], we chose the following four variables to serve as the development metrics for the NFP: (i) the number of people who regularly participate in physical activity, (ii) the passing rate of national physical fitness standards, (iii) the number of public sports venues and facilities, and (iv) the number of social sports instructors. These development metrics serve as dependent variables in the analysis.

This study investigates the socioeconomic factors influencing the development of the NFP. First of all, the sports industry has developed in tandem with public expenditure on culture, tourism, sports, and media (x1). The industry development, in turn, enhances the physical and mental wellbeing of Chinese society [14]. Regardless of sociopolitical structures, the healthcare sector and public services predominantly depend on funding from general public budget expenditure (x2). Likewise, the growth in per capita disposable income (x3) drives the expansion of the sports industry [15]. Generally, when individuals have higher discretionary income, their engagement in sports consumption tends to rise [16], potentially boosting sports participation and physical fitness. Meanwhile, economic progress, such as the GDP expansion (x4), and the sports industry can be viewed as a coupling system [17]. Additionally, the sports industry falls within the tertiary sector, and the value added by this sector (x5) can reflect the development of sports workers and infrastructure [18]. Collectively, these five factors represent the economic-related independent variables.

In terms of social factors influencing the NFP’s development, we analyzed six factors. Population density directly influences participation in leisure-time physical activity. Spatially, East China, representing about one-third of the national population and GDP output, shows a higher prevalence of regular physical activity compared to other regions [19]. Similarly, individuals in urban areas tend to participate in physical activity more frequently than those in rural areas [19]. Hence, the number of residents (x6) can act as a geosocial predictor influencing both the number of people who regularly participate in physical activity (y1) and the passing rate of national physical fitness standards (y2). In market economics, wages are determined by the interplay of supply and demand. The number of social sports instructors (y4) can be influenced by the average wage of sports professionals (x7), especially considering the significant shortage of skilled practitioners in many sectors of the sports industry [20]. Sports-related consumption falls under discretionary expenses and can be influenced by the consumer price index. An increase in the consumer price index may lead to reduced spending on non-essential items. In this context, we investigate the potential impact of the regional consumer price index (x8). An abnormal index could potentially alter consumer behaviors in sports consumption, thereby affecting the development of the NFP. In Chinese contexts, the availability of public transportation can significantly influence the frequency of regular physical activity [21]. Hence, we consider the number of public buses in operation (x9) as a common indicator in our study. Urban areas (x10) and per capita public recreational green space (x11) are suggested factors that influence the physical activity patterns of urban residents. Growing urbanization is often accompanied by increased health consciousness [22], expanded coverage of 15-minute fitness circles and greenway trails [23], and a more advanced regional sports industry [17], all contributing to the advancement of the NFP.

Table 1 outlines the variables utilized in this study. Data pertaining to the development metrics of the NFP can be sourced from provincial and municipal physical fitness monitoring bulletins, while socioeconomic statistics are available in the China Statistical Yearbook. The raw data that support the conclusions of this study are available on figshare (DOI: 10.6084/m9.figshare.25152299.v1).

**Table 1:** Definition of variables.

Type	Variable	Definition	Unit
Dependent variable	y1	Number of people who regularly participate in physical activity	10,000 people
	y2	Passing rate of national physical fitness standards	Percentage
	y3	Number of public sports venues and facilities	Natural number
	y4	Number of social sports instructors	Natural number
Independent variable	x1	Public expenditure on culture, tourism, sports, and media	100 million CNY
	x2	General public budget expenditure	100 million CNY
	x3	Per capita disposable income	CNY
	x4	GDP	100 million CNY
	x5	Value added of the tertiary industry	100 million CNY
	x6	Number of residents	10,000 people
	x7	Average wage of sports professionals	CNY per year
	x8	Regional consumer price index	Index
	x9	Number of public buses in operation	Natural number
	x10	Urban areas	km^2^
	x11	Per capita public recreational green space	m^2^/people

Note. CNY, Chinese yuan.

### 2.2 Spatial analyses

The spatial analyses were conducted using ArcGIS Pro 3.2.1 (Esri Inc., Redlands, CA, USA). Initially, we assessed whether the dependent variables displayed random spatial patterns by calculating the global Moran’s *I*. According to Tobler’s First Law of Geography, “everything is [geographically] related to everything else, but near things are more related than distant things” [24]. Hence, for the computation process, we opted for the common inverse distance method using Euclidean distance metrics. Upon identifying significant spatial autocorrelation, we utilized the Anselin local Moran’s *I* to examine clusters and outliers.

Then, the identified spatial heterogeneity was quantified by fitting regression equations. We fitted a global regression model using ordinary least squares (OLS) after transforming all variables by taking their common logarithmic values to account for measurement dimension differences. The purpose of the OLS analysis was to exclude non-significant variables for the subsequent local regression model, following a criterion-based approach for objective selection [25]. Our initial steps involved conducting a correlation analysis between the dependent and independent variables. Non-significant variables resulting from the correlation analysis were excluded from subsequent analysis. Next, all remaining independent variables were included into the OLS model. We then iteratively removed the independent variable (typically the one with the smallest absolute t-statistic) that yielded the most substantial improvement in the Akaike Information Criterion (AIC) value when eliminated from the model. This process continued until no further removal of independent variables led to a favorable change in the AIC value compared to the previous iteration. At this point, all remaining independent variables were statistically significant, and no issues of multicollinearity (i.e., variance inflation factor < 10) were detected. Effectively, this model selection process argues for a theory that fits the facts (i.e., empirical data), not the contrary.

Subsequently, we fitted a local geographically weighted regression (GWR) model, which can be represented mathematically as Eq (1):

$$y_{i}=\beta_{0}(u_{i},v_{i})+\sum_{j=1}^{n}\beta_{j}(u_{i},v_{i}){x_{i}}_{j}+\varepsilon_{i}$$ (1)

where y_i_ is the dependent variable; *i* is the analyzed variable; *β*_0_(u_i,_ v_i_) is the location of the *i*th variable; *β*_j_(u_i_, v_i_) is the regression parameter, *j*, of the *i*th observation, which is a function of geographical location; x_ij_ is the independent variable; and ε_i_ is the random error of the *i*th dependent variable. At last, the residual of the GWR model was examined using the global Moran’s *I*.

## 3. Results

### 3.1 Spatial autocorrelation

The results of the spatial autocorrelation analysis are summarized in Table 2. Based on the z-scores, only the passing rate of national physical fitness standards (y2) showed significant positive spatial autocorrelation (p < 0.001), while the other dependent variables exhibited complete spatial randomness (p > 0.05). This finding suggests that, in 2021, the passing rate of national physical fitness standards was autocorrelated with the passing rates in nearby regions.

**Table 2:** Global spatial autocorrelation.

Variable	Global Moran’s *I*	z	p
y1	0.074	1.233	0.218
y2	0.379	5.403	< 0.001
y3	0.091	1.391	0.164
y4	-0.014	0.220	0.826

**Fig 1:**
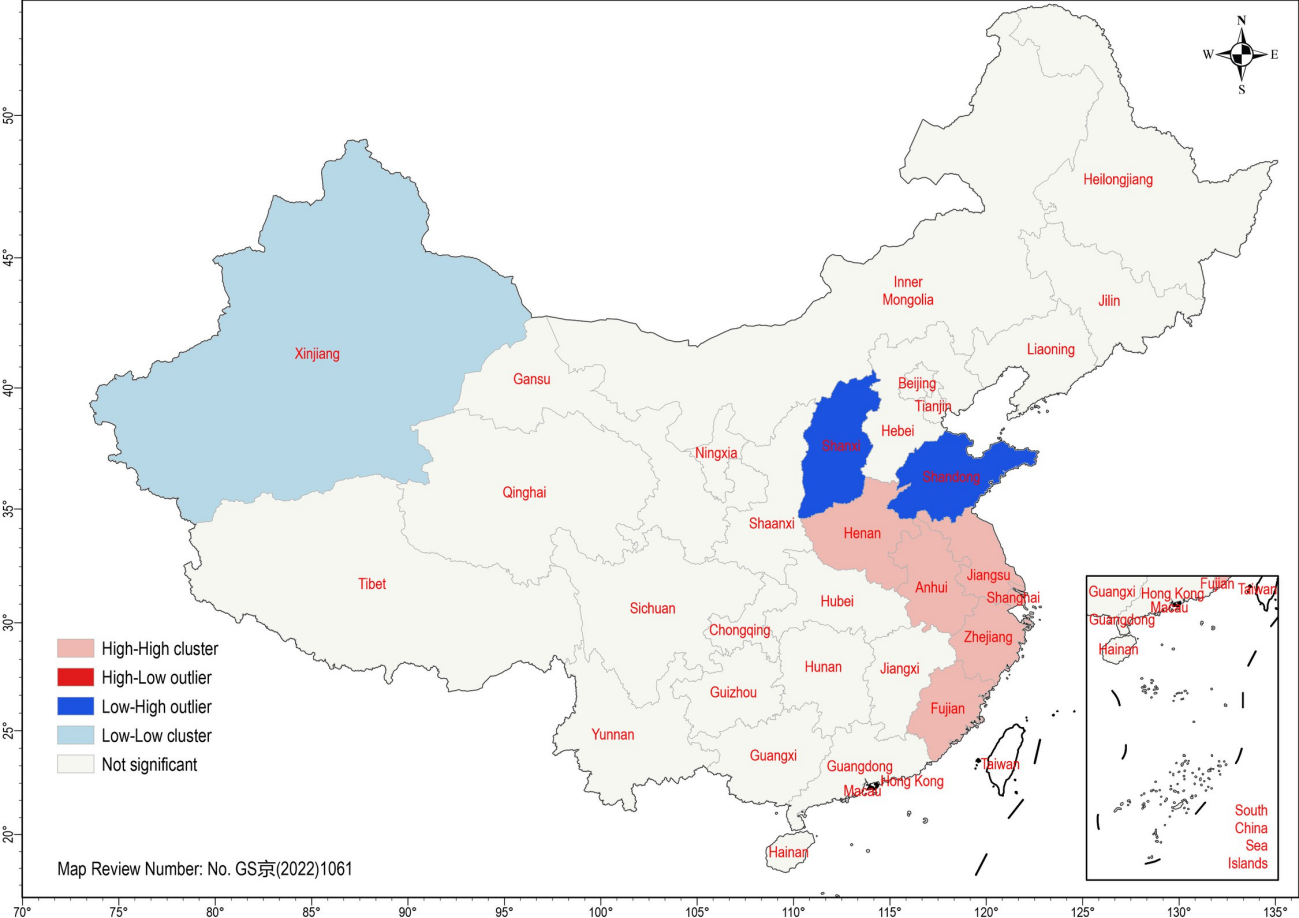
Local spatial autocorrelation analysis of the passing rate of national physical fitness standards.

We proceeded with a local cluster and outlier analysis on y2, as illustrated in Fig 1. The analysis revealed statistically significant z-scores for Xinjiang (z = 4.44), Jiangsu (z = 2.36), Anhui (z = 2.33), Zhejiang (z = 2.23), Shanghai (z = 1.88), Fujian (z = 1.65), Henan (z = 1.62), Shanxi (z = -1.75), and Shandong (z = -2.18) at the p < 0.05 level. These results indicated three distinct spatial patterns. First, Xinjiang formed a low-low cluster of the NFP, surrounded by provinces with low passing rates of national physical fitness standards. Second, spatial heterogeneity was observed in Shanxi and Shandong, suggesting that neighboring provinces’ NFP development was influenced by specific factors. Third, the high-high cluster was predominantly situated in East China, including Jiangsu, Anhui, Zhejiang, Shanghai, and Fujian, indicating a prominent zone for NFP development.

### 3.2 OLS model

Based on the results of the correlation analysis, variables x2, x3, x4, x5, and x9 merit further investigation. Table 3 summarizes the model selection process. We proceeded by removing one variable at a time over four iterations until all three independent variables showed statistical significance without multicollinearity. Surprisingly, x2’s coefficient was negative, indicating that higher general public budget expenditure correlated with a decrease in the passing rate of national physical fitness standards. Attempting to remove x2 in model 3 resulted in a model performance decline, reflected in a 12-point difference in AIC values. Following our model selection criteria, we chose model 3 as the final OLS model. Table 4 presents the regression estimates. Overall, the model was statistically significant (Joint F-Statistic = 15.461) and could explain 63.2% of the variance in the passing rate of national physical fitness standards in 2021. The error term (i.e., p-value of Jarque-Bera Statistic > 0.05) suggests that the residuals of the OLS model were normally distributed, indicating that the model met the statistical assumption.

**Table 3:** Model selection process.

Variable	Model 1		Model 2		Model 3		Model 4	
	t	VIF	t	VIF	t	VIF	t	VIF
x2	-2.94	11.8	-2.98	11.8	-4.10	5.6	-	-
x3	0.88	3.6	1.55	2.6	2.63	1.2	1.85	1.2
x4	-0.82	118.1	-	-	-	-	-	-
x5	0.85	137.6	0.28	29.7	-	-	-	-
x9	3.54	12.2	3.47	11.4	5.30	5.6	2.87	1.2
p	< 0.001		< 0.001		< 0.001		0.0016	
AIC	-102.8		-105.4		-108.4		-96.2	

Note. AIC, Akaike information criterion; t, t-statistic; VIF, variance inflation factor.

**Table 4:** Regression estimates from the OLS model 3.

Variable	Coefficient	SE	p	Diagnostics
intercept	-0.720	0.232	0.004	R² = 0.632
x2	-0.109	0.027	<0.001	Joint F-Statistic = 15.461*
x3	0.062	0.024	0.014	Koenker (BP) Statistic = 6.077
x9	0.094	0.018	<0.001	Jarque-Bera Statistic = 0.713

Note. *p < 0.05.

### 3.3 GWR model

Based on the OLS model, three independent variables—x2, x3, and x9—were included in the GWR model. The z-score of the residuals from the GWR model was 0.026 (p = 0.979), indicating random distribution of residuals. Table 5 summarizes the effect of each independent variable on the passing rate of national physical fitness standards in 2021. The coefficients in the GWR model varied widely across regions. Overall, the model explained 81.8% of the variance in the passing rate, showcasing better model fit than the global model.

**Table 5:** Regression estimates from the GWR model.

Variable	Minimum	Maximum	Median	Mean	SD	Diagnostics
intercept	-0.87	0.083	0.69	0.63	0.204	R² = 0.818
x2	-0.177	0.0244	-0.046	-0.055	0.056	AIC = -120.5
x3	-0.0053	0.086	0.055	0.053	0.0188	Bandwidth = 2942656.4225
x9	-0.0172	0.144	0.0405	0.048	0.047	

Note. The distance band was determined using the Golden search selection method.

**Fig 2:**
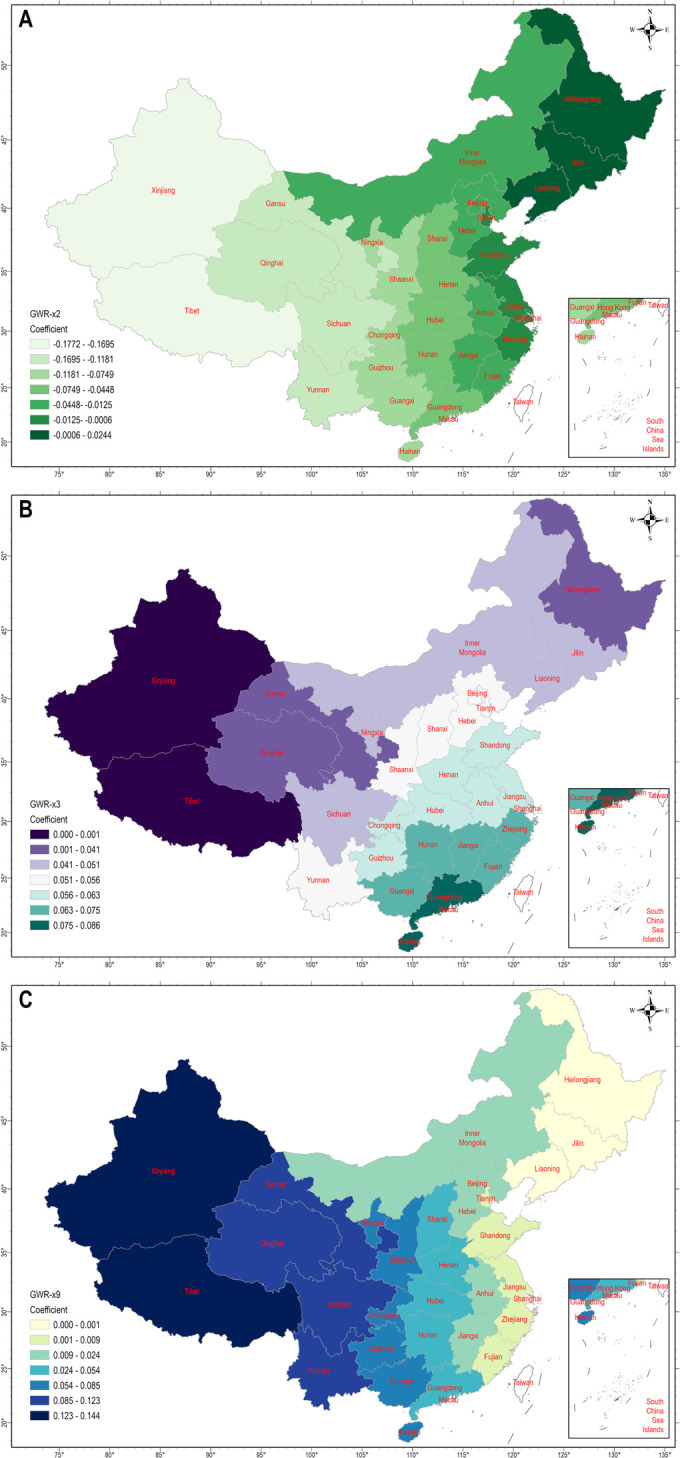
Spatial distribution of GWR regression coefficients: A. general public budget expenditure; B. per capita disposable income; C. the number of public buses in operation.

Fig 2 illustrates the spatial influence of each independent variable on the NFP’s development as of 2021. Detailed statistics are provided in S1 Table. We delve into specific effects of x3 (Fig 2B) and x9 (Fig 2C) in this section, reserving further discussion on x2’s (Fig 2A) negative impact for the next section. Per capita disposable income had a positive effect, albeit diminishing gradually from southeast to northwest regions. Certain regions exhibited lower effects compared to the national average, including Sichuan, Ningxia, Inner Mongolia, Liaoning, Jilin, Heilongjiang, Qinghai, Gansu, Tibet, and Xinjiang. Notably, Tibet and Xinjiang demonstrated minimal influence of per capita disposable income on the NFP. Regarding the influence of public buses, the mean coefficient for this factor, as shown in Table 5, was 0.048. However, individual coefficients varied significantly across the 31 administrative regions, ranging from -0.0172 to 0.144. This variability indicates a substantial regional difference in the impact of public buses on the NFP. Notably, regions like Xinjiang, Tibet, Gansu, Qinghai, Sichuan, and Yunnan relied heavily on public buses, highlighting the importance of the public bus system in Western China’s NFP development. Conversely, coastal regions showed a decreasing influence of public buses.

## 4. Discussion

This study makes two original contributions to the policy evaluation of the NFP. First, we assessed four key metrics of NFP development as of 2021, revealing unequal development in the passing rate of national physical fitness standards. Accordingly, future NFP objectives should prioritize enhancing physical fitness levels in underdeveloped regions of Western China. Second, the GWR model introduces a novel perspective, emphasizing that addressing unequal development requires more than efforts from sports regulators and the sports industry alone. This marks the first-of-its-kind viewpoint on the matter. Both central and local governments must increase fiscal expenditures to build public infrastructures and ensure sustained economic growth for advancing the NFP.

Based on the global Moran’s *I*, there was no regional imbalance observed in the number of people who regularly participate in physical activity, public sports venues and facilities, or social sports instructors. To our knowledge, no previous study has examined the geographic features of the numbers of people who regularly participate in physical activity. A study did, however, identify significant spatial heterogeneity in physical activity participation levels across the 31 administrative regions, although the overall trend indicated a consistent increase from 2010 to 2018 [26]. Taken together, the present result indicates that the nationwide fitness-for-all campaigns in recent years, including the annual National Fitness Day on August 8th, have effectively heightened interest in physical fitness [2]. Similarly, no spatial heterogeneity was found in the number of public sports venues and social sports instructors. The implementation of China’s New Urbanization Plan and Document No. 46 in 2014 notably spurred the growth of the sports industry [17]. Between 2015 and 2021, the annual construction rate of sports facilities surged impressively by 17.48% [17]. These national policies have facilitated the construction of sports facilities not only in affluent areas of East China but also in less developed regions of Western China. However, while the provincial-level data didn’t reveal spatial heterogeneity, disparities exist within cities [27–29] and between urban and rural areas [8]. Addressing these localized disparities requires targeted urban planning strategies.

The passing rate of national physical fitness standards in 2021 exhibited a distinct regional clustering pattern, persisting from the early 2010s [9, 10]. As a matter of fact, the z-score of Moran’s *I* reveals a noticeable increase in clustering from 2014 (z = 2.794) to 2021 (z = 5.403) [9]. This escalation in clustering signifies a deepening divide in the development of national physical fitness standards, highlighting a concerning trend of worsening regional disparities. These findings underscore the critical policy implications addressed by this study.

In 2021, regions in East China exhibited the highest passing rates for national physical fitness standards. Conversely, Northwest China exhibited a noticeable clustering pattern with lower passing rates, while Southwest China as a whole displayed passing rates below the national average. The GWR model sheds light on these regional inequalities through three distinct socioeconomic pathways. First, per capita disposable income was found to potentially promote higher passing rates for national physical fitness standards. GWR coefficients indicated that in 2021, per capita disposable income had minimal impact on national physical fitness in Gansu (β = 0.030), Xinjiang (β = -0.005), and Tibet (β = 0.001). However, this economic factor significantly influenced Central China, particularly in East and Southern China.

Despite ongoing economic inequities, underdeveloped regions in Western China have shown consistent improvement in economic conditions. Government data reveals that Tibet’s per capita disposable income increased from 10,730 CNY in 2014 to 24,950 CNY in 2021. Yet, despite these economic gains, national physical fitness levels in Western China remain stagnant. This suggests a potential threshold for economic development: once surpassed, economic growth may significantly impact national physical fitness. This notion finds support in Niu et al.’s work [30], which explored the association between per capita GDP and public health in 30 administrative regions from 2000 to 2017. Their study identified heterogeneous threshold effects of economic growth, with Central and Western China reaching thresholds at 14,691 and 12,683 USD per capita GDP, respectively. Above these thresholds, economic growth notably influences public health outcomes. In 2021, Tibet’s per capita GDP stood at 8,809 USD, suggesting that despite economic progress, it may not have crossed the critical threshold for substantial impacts on public health. Thus, economic developments, such as per capita disposable income, may not have significantly affected Tibet’s national physical fitness as examined in this study.

Considering the current domestic economic landscape, it is crucial for policymakers to acknowledge the ongoing unequal development of national physical fitness. Projections indicate that this inequality will persist in the foreseeable future. For instance, assuming a 5% annual growth rate in regional GDP, it is estimated that Tibetans will have to wait until 2029 for their per capita GDP to surpass the threshold associated with positive impacts on public health. To align with the physical fitness objectives outlined in the 14th Five-Year Plan, we recommend that the central government takes proactive measures. This includes allocating additional fiscal resources and implementing supportive policies in economically backward regions such as Northeast, North, Southwest, and Northwest China. These initiatives are vital for enhancing GDP output and income growth, ultimately contributing to improved national physical fitness outcomes.

Second, the number of public buses played a critical role in advancing the NFP with a particularly pronounced impact in the expansive territories of Northwest and Southwest China. The correlation between accessible public transportation and increased leisure-time physical activity, enhanced physical fitness, and improved health is evident across various sociodemographic groups [31–33]. The transportation-fitness dynamics were notably accentuated in the Northwest and Southwest regions due to two primary reasons. Geographically, Western China’s intricate topography and diverse climate present challenges to effective urban planning and comprehensive coverage of bus transit networks. Additionally, the absence of subway networks and light rail transit in Western China due to geographical constraints further underscores the reliance on public buses among residents, which in turn affects their engagement in physical activity [34]. This regional disparity in transportation infrastructure can also be attributed to economic development strategies and arguably, a result of human selection. The closer one is to East China, the greater the socioeconomic opportunities, which cannot be denied.

Conversely, it is easy to mistake a coefficient close to zero as indicating a lack of relevance for public transportation overall. According to the GWR coefficients, one might infer that public bus transit in East China did not significantly impact national physical fitness. However, the availability of various transportation options, such as private cars and light rail systems, especially in urban East China, provides more choices. These additional options can offset the reliance on public bus transportation. As studies in developed cities in East China have demonstrated, access to public transportation or convenient and timely access to sports venues and facilities continues to play a significant role in promoting physical activity [35, 36]. Therefore, it will remain a pivotal factor in the development of the NFP.

In our view, there are two policy solutions to enhance access to physical activity. Central and local governments can either increase investment in public transportation or prioritize the establishment of 15-minute fitness circles to address residents’ physical activity needs. We advocate for the latter, especially in Western China. The rationale behind this preference is that while convenient access to sports venues via public transit is important for promoting leisure-time physical activity, the underlying mechanism is the proximity of one’s residence to gyms and the time required to reach them. Wang and colleagues’ study revealed that residents who perceived access to public transport stations within a 10–15 minute walk from their homes were 3.18 times more likely to meet the physical activity guidelines recommended by the WHO [35]. An efficient and cost-effective approach could involve constructing public or privately run gyms within a 15-minute radius of neighborhoods. Initial empirical data support the efficacy of this approach in fostering active lifestyles [13].

Third, it was observed that general public budget expenditure had a negative impact on the NFP. This finding, although unexpected, can be attributed to the general nature of the indicator. According to Chinese legislation, general public budget expenditure encompasses a wide range of expenses such as general public service, foreign affairs, national defense, agriculture, environmental protection, education, science and technology, culture, health, and sports. Therefore, it does not directly measure expenses specifically related to sports. Similarly, public expenditure on culture, tourism, sports, and media (x1) was included in the modeling despite not directly reflecting sports-related expenses. These variables were incorporated based on theoretical considerations of their contribution to public sports services. The negative correlation suggests that the expenditures did not lead to the expected outcomes in public sports services, an issue noted in previous studies [37, 38]. This aspect warrants further investigation.

Due to data availability, the socioeconomic factors examined in this study do not encompass all potential contributors to the NFP’s development. For example, the rising popularity of marathons has led to a notable increase in the participation in running activities [39]. Particularly, the expanding middle class, projected to become the largest social stratum in the future, views marathon running as an ideal activity aligned with their socioeconomic status [40]. This trend is characterized by a heightened awareness of health and wellness. Hence, the number of marathon events could serve as a distinctive metric of the NFP’s impact and may be associated with the passing rate of national physical fitness standards. However, the absence of data precludes the inclusion of this factor in our analysis. Consequently, there is clear room for enhancing the current model in future studies.

The results from China’s national fitness campaign may also hold value for other developing nations seeking to advance public health goals through sports. In our statistical analysis, we employed log transformations on the data to accommodate the varying dimensions of the included variables. This log-log model offers a clear perspective on the magnitude (i.e., coefficient in the regression model) of each contributing variable. In essence, our findings reveal that the impact of public transit in China is just as crucial as economic growth. This implies that making sports venues easily accessible could promote physical fitness. For other developing nations, the key takeaway is that investing in fiscal expenditure or encouraging private investment in close-to-community sports facilities could prove to be an effective strategy for enhancing public health within a constrained fiscal budget.

In conclusion, this study offers an up-to-date assessment of the NFP’s development across 31 administrative regions. Among the NFP’s three key development metrics, only the passing rate of national physical fitness standards in 2021 exhibited spatial heterogeneity. Through the GWR analysis, we identified two significant positive socioeconomic dynamics. From an economic standpoint, the persistent income gap between Western and Eastern China indicates a continued regional imbalance in the NFP’s progress in the foreseeable future. Addressing the socioeconomic disparities affecting NFP development requires comprehensive measures beyond the sports sector. It necessitates proactive involvement from the central government, offering both fiscal support and policy direction. In our view, constructing 15-minute fitness circles, especially in economically backward regions, can be a viable strategy to enhance the NFP participation. Ultimately, these policy suggestions offer fresh perspectives toward achieving the long-range objectives outlined in the 14th Five-Year Plan.

## Supporting information

S1 Table Summary of GWR regression coefficients. (DOCX)
