# Supplementary material for: Empirical analysis of spatial heterogeneity in the development of China’s National Fitness Plan
Source: PLoS One. 2024 Jun 13;19(6):e0305397. doi: 10.1371/journal.pone.0305397 (PMC11175421; doi:10.1371/journal.pone.0305397)
Supplement: S1 Table — (DOCX) [file pone.0305397.s001.docx]

**S1 Table. Summary of GWR regression coefficients.**

| **Province** | **x2** | **x3** | **x9** |
| --- | --- | --- | --- |
| Beijing | -0.012 | 0.052 | 0.014 |
| Tianjin | -0.007 | 0.053 | 0.009 |
| Hebei | -0.016 | 0.053 | 0.017 |
| Shanxi | -0.052 | 0.055 | 0.048 |
| Inner Mongolia | -0.023 | 0.047 | 0.024 |
| Liaoning | 0.014 | 0.047 | -0.009 |
| Jilin | 0.024 | 0.041 | -0.017 |
| Heilongjiang | 0.023 | 0.036 | -0.016 |
| Shanghai | -0.001 | 0.063 | 0.000 |
| Jiangsu | -0.003 | 0.061 | 0.003 |
| Zhejiang | -0.006 | 0.066 | 0.004 |
| Anhui | -0.016 | 0.063 | 0.014 |
| Fujian | -0.014 | 0.071 | 0.009 |
| Jiangxi | -0.028 | 0.069 | 0.022 |
| Shandong | -0.006 | 0.056 | 0.007 |
| Henan | -0.045 | 0.060 | 0.041 |
| Hubei | -0.057 | 0.062 | 0.050 |
| Hunan | -0.063 | 0.066 | 0.053 |
| Guangdong | -0.046 | 0.075 | 0.035 |
| Guangxi | -0.088 | 0.069 | 0.073 |
| Hainan | -0.075 | 0.086 | 0.054 |
| Chongqing | -0.090 | 0.059 | 0.078 |
| Sichuan | -0.118 | 0.051 | 0.102 |
| Guizhou | -0.099 | 0.062 | 0.085 |
| Yunnan | -0.134 | 0.053 | 0.113 |
| Tibet | -0.170 | 0.001 | 0.142 |
| Shaanxi | -0.079 | 0.055 | 0.071 |
| Gansu | -0.120 | 0.040 | 0.106 |
| Qinghai | -0.142 | 0.030 | 0.123 |
| Ningxia | -0.093 | 0.050 | 0.084 |
| Xinjiang | -0.177 | -0.005 | 0.144 |
